# Design, Synthesis and Characterization of a Visible‐Light‐Sensitive Molecular Switch and Its PEGylation Towards a Self‐Assembling Molecule

**DOI:** 10.1002/chem.202201477

**Published:** 2022-07-13

**Authors:** Marco Paolino, Mario Saletti, Annalisa Reale, Mariano Licciardi, Paola Varvarà, Arnaud Marquette, Jérémie Léonard, Claudia Bonechi, Alessandro Donati, Gianluca Giorgi, Germano Giuliani, Benedetta Carlotti, Fausto Ortica, Loredana Latterini, Mariangela Gentile, Eugenio Paccagnini, Massimo Olivucci, Andrea Cappelli

**Affiliations:** ^1^ Dipartimento di Biotecnologie, Chimica e Farmacia (Dipartimento di Eccellenza 2018–2022) Università degli Studi di Siena Via A. Moro 2 53100 Siena Italy; ^2^ Dipartimento di Scienze e Tecnologie Biologiche, Chimiche e Farmaceutiche (STEBICEF) Università degli Studi di Palermo Via Archirafi 32 90123 Palermo Italy; ^3^ Institut de Physique et Chimie des Matériaux de Strasbourg Université de Strasbourg CNRS UMR 7504 Strasbourg France; ^4^ Dipartimento di Chimica, Biologia e Biotecnologie Università di Perugia Via Elce di Sotto, 8 06123 Perugia Italy; ^5^ Dipartimento di Scienze della Vita Università degli Studi di Siena Via A. Moro 53100 Siena Italy; ^6^ Chemistry Department Bowling Green State University 43403 Bowling Green OH USA) ok

**Keywords:** HBDI-like chromophores, light-driven molecular switches, light-sensitive molecules, nanoaggregates, photoswitches, self-assembling molecules

## Abstract

HBDI‐like chromophores represent a novel set of biomimetic switches mimicking the fluorophore of the green fluorescent protein that are currently studied with the hope to expand the molecular switch/motor toolbox. However, until now members capable of absorbing visible light in their neutral (i. e. non‐anionic) form have not been reported. In this contribution we report the preparation of an HBDI‐like chromophore based on a 3‐phenylbenzofulvene scaffold capable of absorbing blue light and photoisomerizing on the picosecond timescale. More specifically, we show that double‐bond photoisomerization occurs in both the *E*‐to‐*Z* and *Z*‐to‐*E* directions and that these can be controlled by irradiating with blue and UV light, respectively. Finally, as a preliminary applicative result, we report the incorporation of the chromophore in an amphiphilic molecule and demonstrate the formation of a visible‐light‐sensitive nanoaggregated state in water.

## Graphical abstract

**Exploiting the conjugation** of the 3‐phenyl benzofulvene scaffold, we prepared a new HBDI‐like chromophore capable of absorbing blue light and photoisomerizing in the picosecond timescale. The PEGylation of this photoswitch lead to a visible light‐sensitive amphiphilic molecule capable of self‐assembly in water to give a spherical nanoaggregated structure.

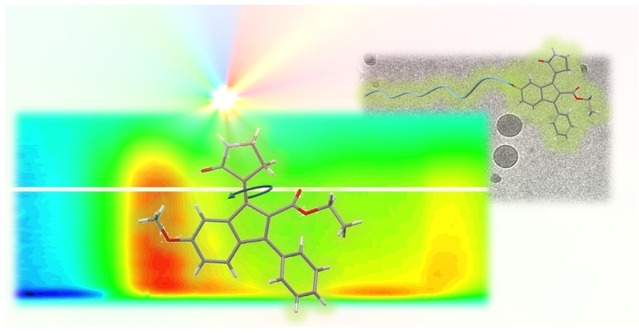

## Introduction

Light‐driven molecular switches (LDMSs) are molecules capable of interconverting between at least two different isomeric forms characterized by distinct absorption spectra as a result of a light stimulus.^[1]^ Nature, more perfectly than humans, uses LDMSs to regulate numerous biological processes.^[2]^ Natural light‐sensitive proteins, such as Rhodopsins and Photoactive Yellow Protein (PYP), provided a template for the design of increasingly efficient synthetic LDMS as molecular elements for nanodevices and nanomedicines.[^3^, ^4^, ^5^, ^6^] In the last decade, many classes of molecules (i. e. azobenzenes,[^7^, ^8^] spiropyrans,[^9^, ^10^] stilbenes,^[11]^ diarylethenes,^[12]^ indigoids,^[13]^ etc.) have been designed for the conversion of light in mechanical motion of isolated molecules[^14^, ^15^] but also in 2D ordered supramolecular structures.[^16^, ^17^] Among these, overcrowded olefins capable of rotation around the C=C double bond showed interesting properties and promising applications.[^18^, ^19^] In these structures, light energy fuels the rotation of a portion of the molecule (rotor) with respect to a reference fixed portion (stator) via a *cis to trans* and/or *trans to cis* isomerization of an exocyclic double bond.^[20]^ Numerous studies aim to control the rotational speed,^[21]^ modulate the absorption wavelength,[^22^, ^23^] increase the quantum yield of isomerization,[^24^, ^25^] increase stability (showing increased numbers of photocycles)[^26^, ^27^] or unidirectional movement (evolution from photoswitches to light‐driven molecular rotors),[^28^, ^29^] and to expand the solubility in different organic and aqueous solvents in these systems.[^30^, ^31^, ^32^]

**Figure 1:**
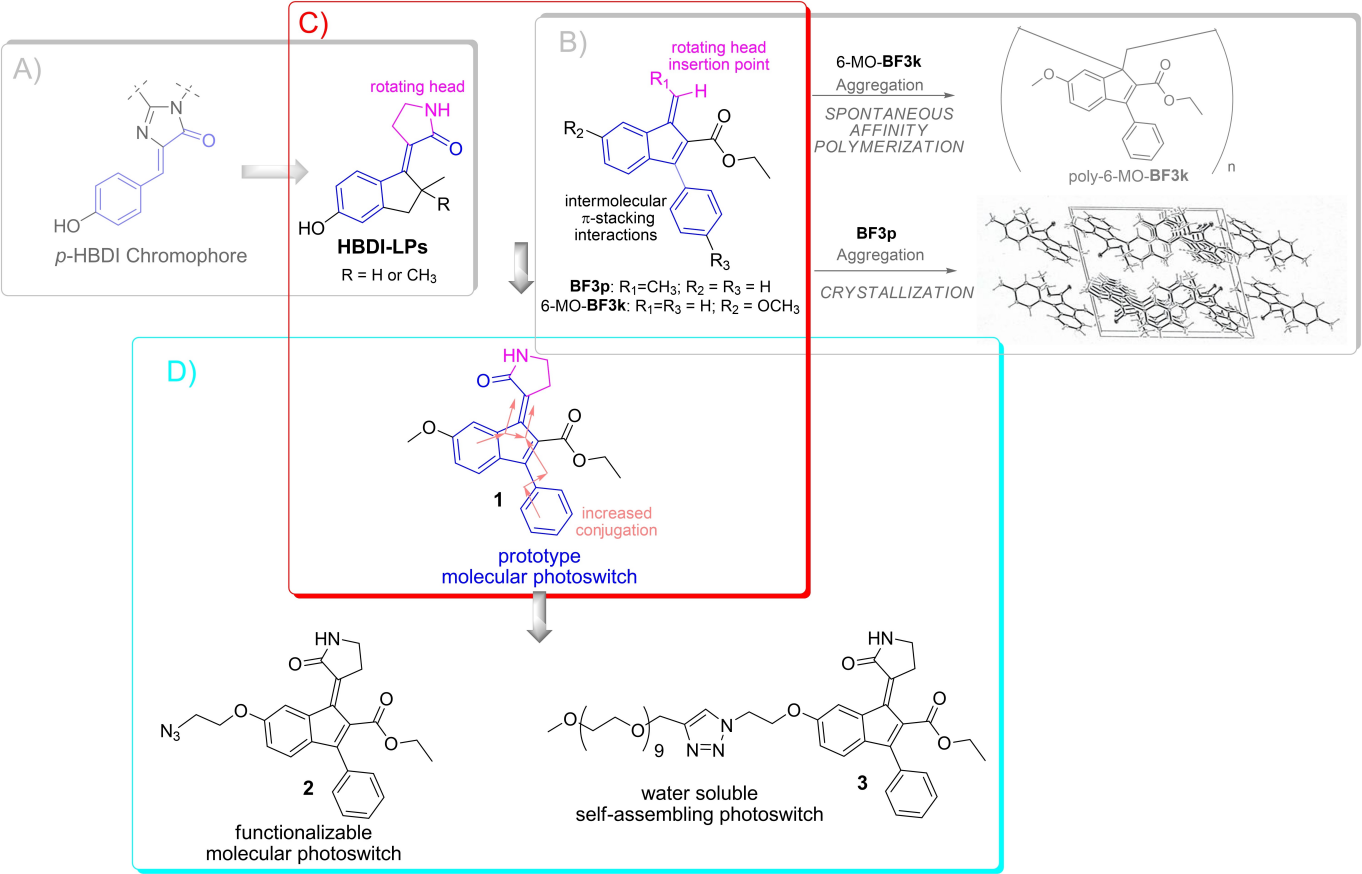
Design of the new LDMS **1** from GFP fluorophore through HBDI‐like photoswitch and its rational conversion in the functionalizable LDMS **2** and pegylated water‐soluble self‐assembling LDMS **3**.

Over the past decade, part of our work has been focused on the design, synthesis, and computational characterization of new LDMS. In particular, we have successfully tried to mimic the reactivity of the retinal chromophore embedded in the Rhodopsin cavity. To this scope, we have prepared analogues positively charged N‐alkylated or N‐protonated indanylidene pyrroline Schiff bases (NAIPs and NHIPs respectively), which perform ultra‐fast double bond photoisomerization.[^33^, ^34^, ^35^, ^36^, ^37^] The same biomimetic approach was then used to design a negatively charged LDMS.[^38^, ^39^] In this last design effort, we were inspired by the structure of the Green Fluorescent Protein (GFP) fluorophore 5‐(4‐hydroxybenzylidene)‐1*H*‐imidazol‐2(5*H*)‐one (*p*‐HBDI), to transform an efficient emitter into an LDMS that featured a sub‐picosecond *E*/Z photoisomerization (**HBDI‐LPs**, see Figure 1A) under UV light exposure.[^38^, ^39^]

In parallel, some of us have developed the concept of “affinity polymerization” based on the molecular recognition between 3‐phenylbenzofulvene derivatives (i. e. 6‐MO‐**BF3k** ^[40]^ reported in Figure 1C) mediated by intermolecular π‐stacking interactions.[^41^, ^42^, ^43^, ^44^] In these systems, spontaneous polymerization occurs in the apparent absence of catalysts or initiators and involves the exocyclic double bond of these *trans*‐diene monomers. However, the introduction of a methyl group on the exocyclic methylene (i. e. **BF3p**) prevents spontaneous polymerization while allowing the formation of columnar aggregates in the solid‐state.^[45]^ Interestingly, the through‐space conjugated monomeric units of these polybenzofulvene derivatives showed emissive properties and the ability to transfer the absorbed energy to small molecule guests by fluorescence resonance energy transfer (FRET) mechanism.[^44^, ^46^, ^47^, ^48^, ^49^, ^50^, ^51^]

In the present paper, we report a novel HBDI‐like homologue photoswitch capable of functioning in both the *E*‐to‐*Z* and *Z*‐to‐*E* directions via visible and UV light, respectively. More specifically, **1** features an extended conjugation due to a 3‐phenylindene‐2‐carboxylate scaffold, the structure at the basis of the polybenzofulvene monomeric unit (Figure 1C).[^52^, ^53^, ^54^, ^55^, ^56^, ^57^, ^58^, ^59^, ^60^, ^61^, ^62^, ^63^, ^64^, ^65^] We also show that the obtained LDMS **1** can be easily functionalized with a clickable azide group (compound **2**) for the insertion of the photoswitch in more complex synthetic and/or biological structures via azide‐alkyne click reactions. In fact, we report that methyl‐end‐capped nona(ethylene glycol) (NEG) side chain, an amphiphilic solubilizing group,^[66]^ can be successfully conjugated with **2** in a preliminary effort to obtain an amphiphilic LDMS capable of self‐assembling in nanometric micelles in water solution and operated by visible light (Figure 1D).

## Results and Discussion

### Design and Synthesis

Recently, our research team reported on the synthesis and characterization of molecular photoswitches inspired by the chromophore of GFP.[^38^, ^39^] These photoswitches (**HDBI‐LPs** in Figure 1, panel A) share with the natural GFP and PYP^[67]^ chromophores, the cinnamic structure characterized by an aromatic ring connected by means of an exocyclic double bond to a carbonyl group. As introduced above, the cinnamic structure is also present in another chemical scaffold widely studied in our laboratories, namely ethyl 1‐methylene‐3‐phenyl‐1*H*‐indene‐2‐carboxylate, which we will call benzofulvene scaffold hereafter.^[68]^ In this structure, the double bond of the cinnamic scaffold is harnessed in the 5‐term ring without the possibility of isomerization. However, as in **HDBI‐LP**, also in the benzofulvene scaffold there is an exocyclic double bond, which, in the dependence of the appropriate functionalization, can lead to the formation of distinct configurational isomers. Therefore, the benzofulvene structure of compound 6‐MO‐**BF3k** ^[40]^ (Figure 1, panel B) was used to insert the rotating head of **HDBI‐LP** to obtain the newly designed photoswitch prototype **1**, in which two aromatic rings are conjugated with the amide carbonyl by means of a diene moiety, which extends the electronic conjugation with respect to that of the previous **HDBI‐LP**.

Among the benzofulvene structures studied in our laboratories, the **BF3k** scaffold bearing a methoxy group in position 6 of the indene nucleus (i. e. 6‐MO‐**BF3k**) was selected due to its tolerance to synthetic structural manipulation. The presence of the oxygen atom in position 6 was then exploited to introduce a clickable azide group without altering the chromophore structure as in compound **2** (Figure 1, panel C). A first example of compound **2** functionalization is represented by amphiphilic compound **3** (Figure 1, panel C), obtained by conjugation with a chain of methyl‐end‐capped nona(ethylene glycol) (NEG). The synthetic efforts and the characterization of the compounds are accurately described in the Supporting Information and reported in Schemes S1–S3.

**Figure 2:**
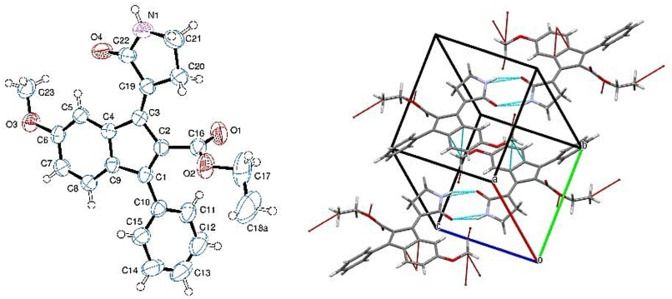
Left panel: Structure of the major isomer of compound 1 obtained by crystallographic studies. Ellipsoids enclose 50 % probability. Right panel: H‐bond interaction involving the pyrrolidinone moiety and short contacts in the crystal of model compound **1**.

Particular attention has been paid to the crystallographic structure of compound **1**, which allows us to unequivocally assign the *E* configuration to the preponderant diastereomer, and gives the opportunity to observe relevant intermolecular H‐bond interactions in the crystalline packing occurring between the pyrrolidinone heads (Figure 2).

### Photophysical and photochemical features of the novel chromophore

**Figure 3:**
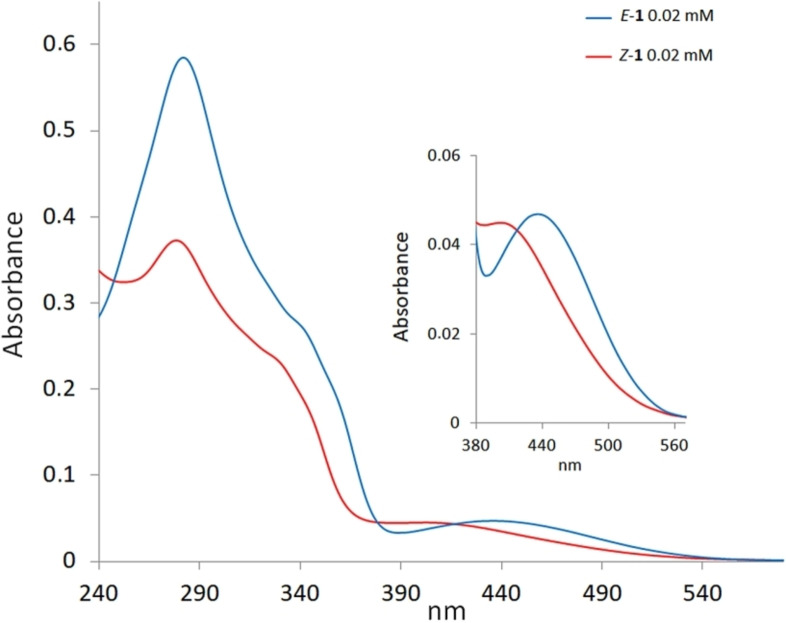
Comparison of the UV‐vis spectra of *E*‐**1** (blue lines) and *Z*‐**1** (red lines) registered at 0.02 mM in methanol.

The initial photophysical and photochemical characterization focused on the *E/Z* diastereoisomers of **1**. The absorption spectrum of *E‐* **1** in methanol (Figure 3) is composed of a main band centered at 282 nm, a shoulder at 340–360 nm and a weaker band centered at 435 nm and spanning up to about 550 nm. Similar absorption spectra were obtained both in chloroform and dichloromethane. However, in these aprotic solvents, despite an apparently rapid dissolution of the crystals, a few minutes are required to get *E‐* **1** molecularly dissolved (Figure S1). The difference in the dissolution speed may be due to the presence of solute‐solute (intermolecular) hydrogen bonds (as suggested by the crystallographic studies, see Figure 2), which could be rapidly broken in methanol. Similar optical features were observed in the UV‐vis spectra of *Z*‐**1**. However, both components of the UV‐vis spectra of *Z*‐**1** are blue‐shifted and with a lower absorbance with respect to the spectra of *E*‐**1**. This blue‐shift effect is particularly evident in the visible region, in which the *Z*‐**1** absorption maximum is 30–35 nm shifted with respect to *E*‐**1** (Figure 3, inset).

For comparison, the visible component in the absorption spectra of model compound **1** is absent in those of the original **HBDI‐LPs** consistently with the increased conjugation. Moreover, as expected, the UV‐visible absorption features of clickable compound *E*‐**2** are similar to those of **1** supporting the basic design assumptions (Figure S3).

**Figure 4:**
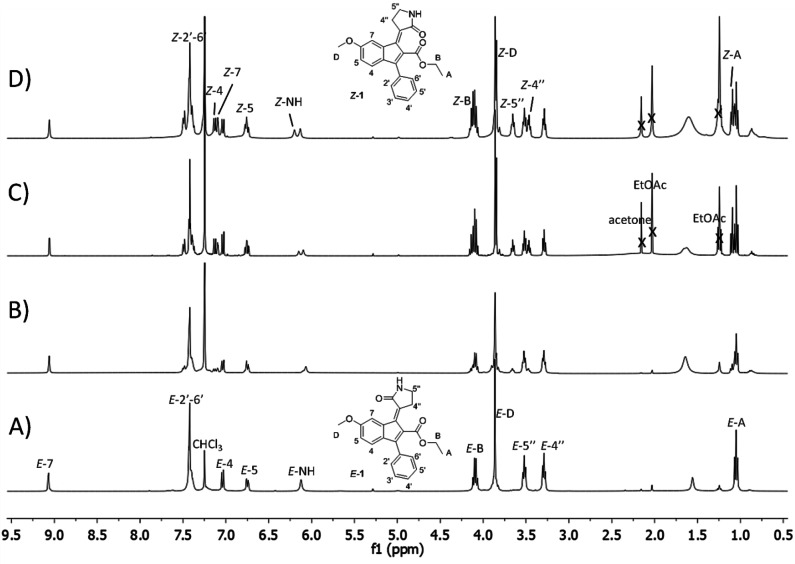
Comparison of the ^1^H NMR spectra (400 MHz, CDCl_3_) of prototype molecular photoswitch *E*‐**1** (A) with the mixture of the *E* and *Z* isomers at the PSSs reached after irradiation at 320 nm (B), 380 nm (C), and 440 nm (D).

Based on the UV‐vis absorption spectra, the photoisomerization of **1** was evaluated by irradiating its CDCl_3_ solution into a Pyrex NMR tube at room temperature with three different wavelengths each corresponding to one of the described bands. The results are shown in Figure 4.

The *E*/*Z* ratio of the photostationary states (PSS) of **1** was determined using ^1^H NMR spectroscopy by computing the area ratio of the well distinguishable *E* ad *Z* signals. The *Z* isomer proportion of the PSS composition increased from 33 % to 43 % and 49 % when increasing the excitation wavelength from 320 nm, to 380 nm and 440 nm, respectively. This behaviour the PSS allows to modulate the *E*/*Z* composition in solution through the UV and visible light. The solutions at the PSSs were stored at room temperature in the dark for a few days without displaying a significant change in *E*/*Z* composition suggesting the presence of a considerable interconversion thermal barrier between the two isomers. Moreover, high thermal stability (without any isomerization or decomposition) was confirmed for *E*‐isomer by means of ^1^H NMR spectroscopy after heating at 50 °C in deuterated methanol or at 100 °C in DMSO‐d_6_ for 24 h.

**Figure 5:**
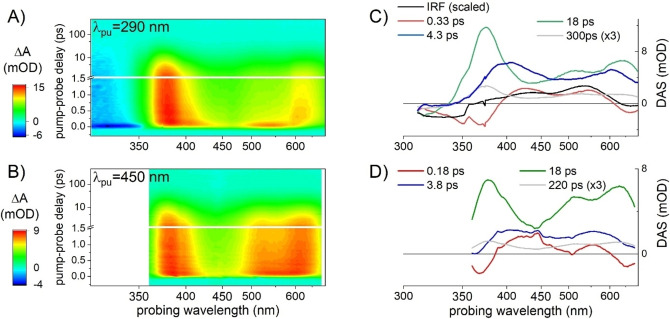
Transient absorption data (ΔA, coded in flase‐color scale) as a function pump‐probe delay (ps) and probing wavelength (nm), recorded on a dichloromethane solution of *E*‐**1** upon excitation at (A) λ_pu_=290 nm and (B) λ_pu_=450 nm, and the corresponding decay associated spectra (DAS) resulting from their global analysis (C) and (D), respectively.

**Figure 6:**
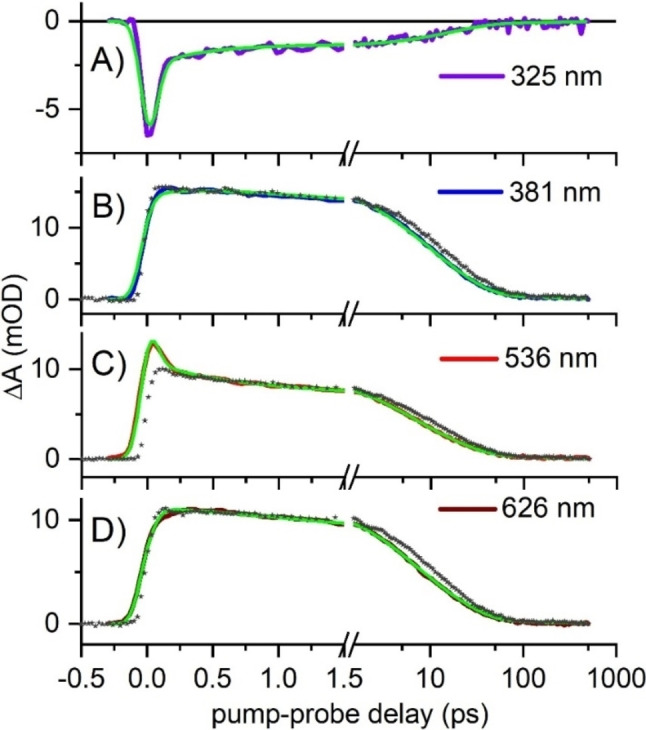
Selection of kinetic traces recorded for a dichloromethane solution of *E*‐**1** upon excitation at λ_pu_=290 nm (solid lines) and their global fit (in green), for a selection of probing wavelengths. In panels B to D, the kinetic traces recorded at the same probing wavelengths for λ_pu_=450 nm (grey stars) are overlapped (after normalization), showing that the only differences are (i) in the 5 to 10 ps range where the decay appears slightly slower when λ_pu_=450 nm, and (ii) in panel C, where the overshoot around time zero for λ_pu_=290 nm is the very short‐lived signature of the S_X_ ESA observed before internal conversion to S_1_.

To gain insight into the photoreaction dynamics of **1**, we performed transient absorption (TA) spectroscopy (see Experimental Section/Methods) on two solutions of *E*‐**1** dissolved in dichloromethane at a concentration of 1.9 mM, in two experiments using a pump pulse tuned at λ_pu_=450 nm or λ_pu_=290 nm. For the latter experiment, the probing detection window was specifically extended down to 320 nm (see Experimental Section/Methods) in order to monitor the recovery of the ground state bleach (GSB). Both datasets are displayed in Figure 5A and 5B, and reveal a very similar, picosecond‐lived induced absorption (ΔA>0 coded in green to red) with local maxima around 375 nm, 510 nm and 640 nm. This spectrally very broad induced absorption is assigned to the S_1_ excited state absorption (ESA) since it is observed when exciting at λ_pu_=450 nm (Figure 5B), i. e. in the weak, low‐energy absorption band of *E*‐**1**. With λ_pu_=290 nm, we excite *E*‐**1** in its stronger UV absorption band and populate a higher‐lying S_X_ electronic state (X>1), which is characterized by an ESA band centred around 530 nm and observed only around time zero in Figure 5A and 6C. This initial S_X_ ESA disappears within the instrument response function to give rise to the S_1_ ESA, indicating ultrafast internal conversion from the initial S_X_ state to S_1_ on a time scale faster than the experimental time resolution (∼60 to 80 fs). The initial GSB signal (ΔA<0) observed at λ<350 nm in Figure 5A and 6A decays on the same sub‐80 fs time scale, which we interpret as due to the formation of the S_1_ ESA which would extend also in the UV to λ=310 nm or shorter wavelengths and thus spectrally overlap and partially cancel the GSB signature. Following this initial internal conversion from S_X_ to S_1_, the kinetics of the S_1_ ESA decay is almost identical in both datasets as illustrated for a selection of probe wavelengths in Figure 6.

The quantitative analysis of both datasets is performed independently by global analysis (see Experimental Section/Methods). The results are illustrated by the decay‐associated spectra (DAS) displayed in Figure 5C and 5D for λ_pu_=290 nm and λ_pu_=450 nm, respectively. Besides the spectroscopic signature characterizing the ultrafast, non‐resolved decay of the S_X_ signature (black line “IRF” in panel C), the decay‐associated spectra (DAS) are nearly identical in both experiments. In particular an early spectral relaxation is observed in S_1_ within 0.33 and 0.17 ps for λ_pu_=290 nm and λ_pu_=450 nm, respectively, corresponding to the blue shift of the 375 nm band and red shift of the 640 nm band of the broad S_1_ ESA (red DAS in Figures 5C and 5D). Then the S_1_ ESA decays on two dominating time scales of 3.8 to 4.3 ps and 18 ps (blue and green DAS in Figures 5C and 5D, respectively, and a minor time scale of 200–300 ps (grey DAS, scaled x3 in Figures 5C and 5D). The shapes of these DAS hardly depend on the pump wavelength, and the 18 ps and 200–300 ps DAS have almost identical shapes. We note that the 18 ps DAS becomes negative at wavelengths λ<340 nm which clearly reveals the GSB recovery – i. e. S_1_ to S_0_ decay – on this time scale. The 4 ps DAS is nearly vanishing in the same spectral region where we already noticed that GSB and S_1_ ESA signatures overlap and partially cancel out. Hence, we argue that the very weak amplitude of the 4 ps DAS for λ<340 nm is the result of the concomitant GSB recovery (negative decay amplitude) and ESA decay (positive decay amplitude). Altogether, we propose that the 4 ps and 18 ps DAS characterize the bi‐ (multi‐) exponential decay of two (or a distribution of) S_1_ subpopulations.

In addition, we note that the relative amplitude of the 4 ps (green) DAS relative to the 18 ps (blue) DAS is larger for λ_pu_=290 nm as compared to λ_pu_=450 nm. In the former case, we expect that the higher photon energy results in a globally hotter S_1_ population produced upon ultrafast internal conversion from S_X_. Hence, we propose that the 4 ps decay component characterizes a vibrationally hotter subpopulation while the 18 ps lifetime would correspond to cooler molecules. Finally, the 220 to 300 ps decay time scale would correspond to a minor, vibrationally relaxed S_1_ population which would decay to S_0_ by overcoming an S_1_ energy barrier trapping this subpopulation over few hundred ps.

To summarize, the reaction kinetics revealed by the S_1_ spectral relaxation and triexponential decay kinetics observed upon excitation at both wavelengths is compatible with a slowing down of the S_1_ population decay – suggesting that the S_1_‐to‐S_0_ decay region of the conformational space becomes less and less accessible – while the vibrational relaxation proceeds in S_1_. Finally, at the longest time delays (500 ps) achieved in this experiment, a very weak signal persists which still displays a spectral signature very similar to the 18 ps and 200 to 300 ps DAS representative of the S_1_ ESA. In particular, no *Z‐E* difference spectrum clearly emerges suggesting that the *E*‐to‐*Z* photoisomerization quantum yield (QY) is small. This observation is confirmed by the independent determination of the photoisomerization quantum yield, revealing that QY<0.1 % (see Experimental Section/Methods).

As compared to the related **HBDI‐LP** compounds,[^38^, ^39^] the modified electronic structure of *E‐* **1** (increased π electron conjugation) results in (i) a significant reduction of the S_0_‐S_1_ energy gap and oscillator strength, evidenced by the weak absorption band around 435 nm as compared to λ_max_=320 nm for **HBDI‐LP**, (ii) a significantly longer excited state lifetime reaching and exceeding the 10 ps time scale (vs. sub‐ps to ps for **HBDI‐LPs** in its anionic[^38^, ^39^] or neutral (unpublished) forms), indicating a larger energy gap or barrier between the S_1_ minimum and the conical intersection, and (iii) a strongly reduced photoisomerization quantum yield respect to the anionic **HBDI‐LPs** (QY=17–19 %).[^38^, ^39^]

### Photophysical features of the chromophore in the amphiphilic compound

**Figure 7:**
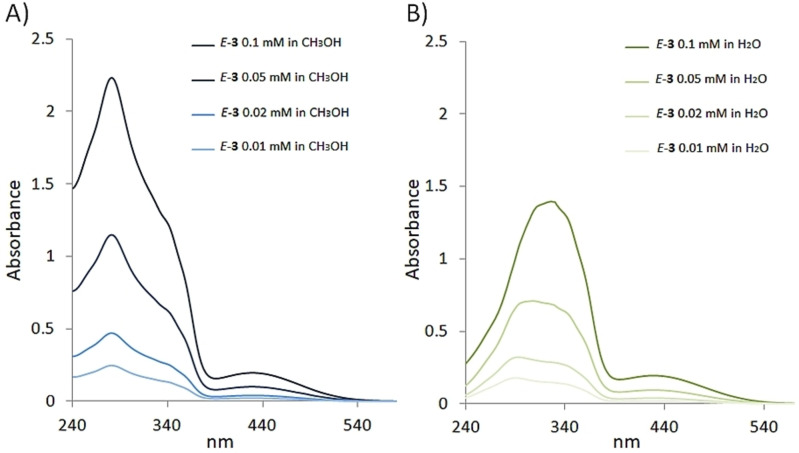
Comparison of the UV‐vis absorption spectra of the compound *E‐* **3** in methanol (A), and water (B) at concentrations ranging from 0.1 to 0.01 mM. The methanolic solution of compound *E*‐**3** follows the Lambert‐Beer law, however the spectra at different concentrations in this solvent are shown for a direct comparison with those recorded in water.

Owing to its amphiphilic character, the photophysical features of **3** were investigated both in water and methanol. The methanol solutions of *E*‐**3** showed absorption spectra (Figure 7) very similar to that of *E*‐**1** (Figure 3), suggesting the presence of isolated chromophores, rather than an aggregate, in solution in the tested concentration range (0.1 mM–0.01 mM). On the other hand, the absorption spectra of PEGylated compound *E*‐**3** in water (Figure 7) are affected by the concentration in the range 0.1–0.01 mM suggesting the possibility of aggregate formation. At the highest concentration tested, the UV‐vis spectrum shows an intense band centered at about 325 nm and a weaker and wider one centered at 430 (spanned up to about 550 nm). A progressive blue‐shift of the main band in the UV range was observed with the decrease of the concentration.

**Figure 8:**
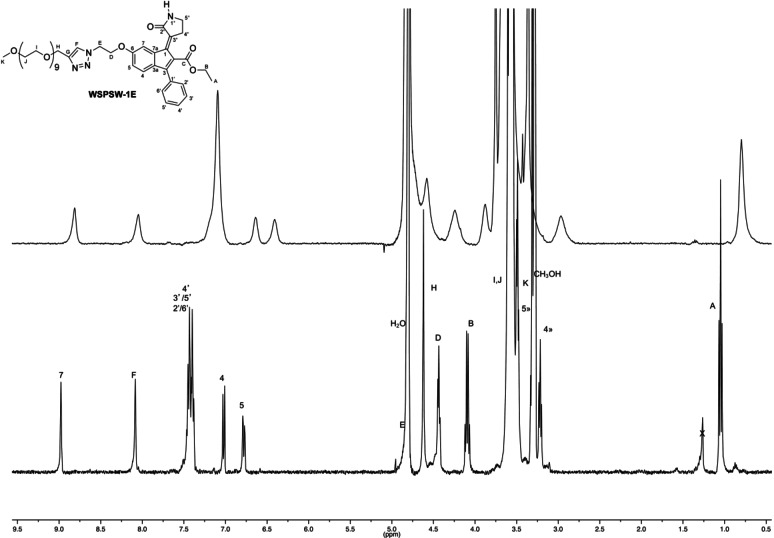
Comparison of ^1^H NMR spectrum (400 MHz) of *E*‐**3** in deuterated water (top trace), CD_3_OD (bottom trace).

To further investigate the aggregation behaviour, the ^1^H NMR spectra of compound *E*‐**3** were performed in D_2_O and CD_3_OD (Figure 8). The spectrum of the water solution showed much broader signals than in methanol‐*d_4_* and deuterated chloroform suggesting the aggregation propensity of *E*‐**3** in water environment.

**Figure 9:**
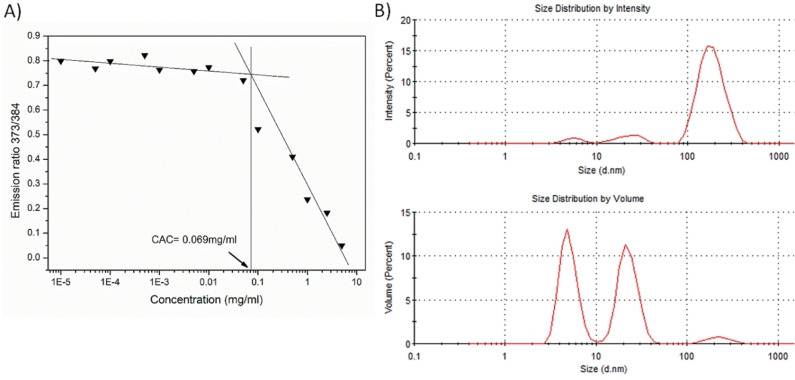
A) I_373_/I_384_ intensity ratio obtained from pyrene emission spectra in the presence of pure *E*‐**3** as a function of the logarithm of its concentration in water. B) DLS size distribution histograms (by intensity and volume) of *E*‐**3** dispersions in bidistilled water at concentration values of 0.1 mg/mL.

Thus, the aggregation features of *E*‐**3** in water was investigated by means of different approaches such as pyrene fluorescence analysis, dynamic light scattering (DLS), and transmission electron microscopy (TEM and CryoTEM) techniques.^[69]^ Fluorescence analysis with pyrene as the probe allowed us to determine a critical aggregation concentration (CAC) value of 0.07 mg/ml as reported in Figure 9A suggesting high aggregation liability for *E*‐**3** in water. The transparent solutions of water‐soluble derivative in bidistilled water were analyzed by dynamic light scattering (DLS). DLS analysis confirmed the propensity of *E*‐**3** to generate several heterogeneous populations nanoaggregates in water at the concentrations above its CAC (see Figure 8B). The most stable population showed small mean diameters (from 3 to 10 nm) and were assumed to be composed by small aggregates. On the other hand, the other components showed greater dimensions (around 30 and 200 nm) and were obviously constituted by a large number of molecules.

**Figure 10:**
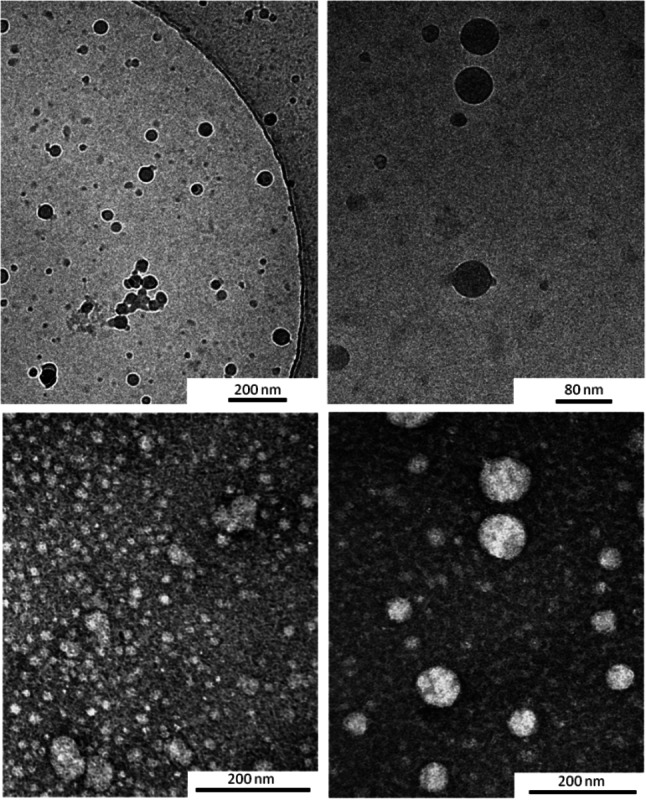
CryoTEM image (up panels) and TEM image (bottom panels) obtained with a water solution of **3** at a concentration of 10 mg/mL, i. e. well above its CAC.

The cryoTEM investigations performed on a 10 mg/mL water solution (i. e. at a concentration well above its CAC) confirmed the presence of heterogeneous populations of spherical particles with a large preponderance of small particles showing dimensions in the range 10–80 nm (Figure 10). Similar results were obtained by TEM investigations performed by negative staining with uranyl acetate on the same sample as used in cryoTEM studies confirming the presence of heterogeneous populations of spherical particles with a large preponderance of small and very small particles (Figure 9).

### *E* to *Z* photoisomerization via ambient light irradiation

**Figure 11:**
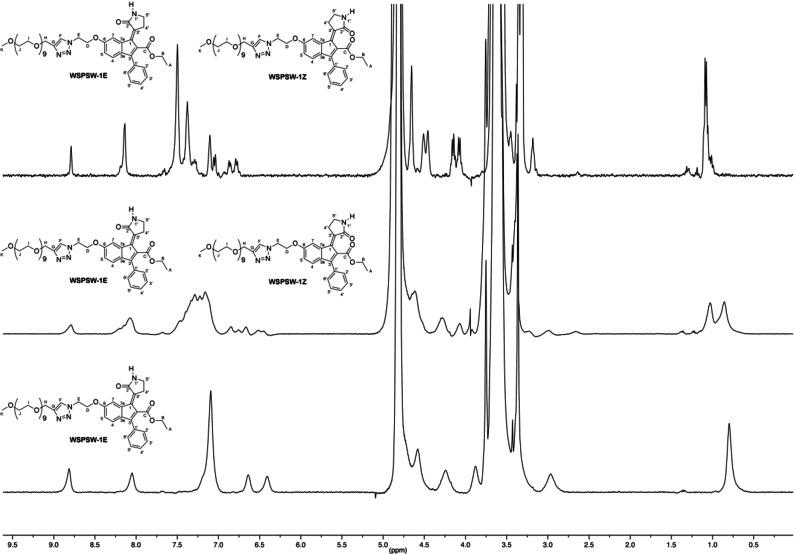
Isomerization of compound *E*‐**3** in deuterated water at ambient conditions. Comparison of ^1^H NMR spectrum (400 MHz) of *E*‐**3** in deuterated water (bottom trace) with that of the same solution obtained after four months storage at ambient conditions (middle trace). The top trace represents the spectrum obtained by addition of methanol to the water solution in order to break the aggregates; methanol was added immediately before of recording the spectrum.

Owing to the presence of a significant absorption band in the visible region, we placed our attention on the interaction of compound **3** with the visible light by comparing its behavior in water and methanol solutions. When water and methanol solutions of pure compound *E‐* **3** were analyzed by ^1^H NMR spectroscopy after some weeks of storage at ambient conditions (i. e. room temperature and indoor light irradiation), interesting differences were observed involving the photoisomerization process in the two samples. In particular, the ^1^H NMR spectrum obtained with the sample dissolved in deuterated water showed the presence of almost equivalent amounts of the two isomers (Figure 11), whereas in the spectrum obtained with methanol solution we observed, along with the signals attributed to *E*‐**3** a set of small signals attributed to slight decomposition. For comparison, a similar monitoring was also performed on *E*‐**1** in methanol and no photoisomerization was observed for prolonged exposure to room light.

Thus, a kinetic study was performed to further evaluate the effect of indoor light irradiation on the *E*‐**3** solution in deuterated water. Specifically, two samples were prepared in two 5 mm NMR tubes and kept in the lab conditions, but one was kept carefully protected from the indoor light by using an aluminum film around the NMR tube. The results of these experiments supported the key role of the indoor visible light in triggering the photoisomerization process, which was not operative in the absence of light and led to an almost complete inversion of the configuration at the exocyclic carbon‐carbon double bond after 17 weeks.

These observations confirmed that the compound *E*‐**3** was susceptible of isomerizing in the ambient conditions and the isomerization process could be affected by environment features such as solvent[^70^, ^71^] or, more interesting, the aggregation state[^72^, ^73^] which can influence the energy barrier of the double bond interconversion.

## Conclusion

In summary, we have designed a new generation of our LDMS, in which the structure of the original compound **HBDI‐LP** has been manipulated. In particular, the chromophore conjugation was extended in the aim of shifting the absorption spectrum in the visible range, and a clickable azide group was introduced without altering the chromophore structure. The resulting compounds **1**–**3** showed the expected photophysical features with an absorption tail spanning up to 550 nm in the visible range. Photoisomerization studies performed on **1** showed that the *Z* isomer composition of the PSS increases with the increase of the illumination wavelength, allowing the *E*/*Z* composition to be modulated in solution through alternative UV or blue light irradiation. TAS analysis revealed that the S_1_ ESA decay is multiexponential with dominating time constants of 4 ps and 18 ps. We propose that the S_1_ decay slows down while the S_1_ population cools down, indicating the presence of an excited state energy barrier (or extended flat region) trapping the vibrationally relaxed S_1_ subpopulation. This excited state dynamics is very different from that of the **HBDI‐LP**, where the S_1_ population decays on a the ps to sub‐ps time scale, that is. before complete vibrational relaxation and cooling.^[38]^

Interestingly, compound **3** appeared to photoisomerize when exposed to the indoor visible light of our lab in a process affected by the solvent, being more pronounced in water than in methanol. This difference should be related to the solvent and aggregation features since **3** appeared to be molecularly dissolved in methanol, but strongly aggregated in water. In these aggregates, the amphiphilic oligo(ethylene glycol) chains‐tails are probably located in the aggregate edge and exposed to the water environment, while the relatively hydrophobic chromophores would be preferentially located inside the core of the micelle. In this organization, the intermolecular H‐bond interactions seen in the solid – state of compound **1** could be conserved and can lead to the formation oheterogeneousus supramolecular species governed by H‐bonds in a hydrophobic shell stabilized by the well‐know intermolecular π‐stacked interactions derived from the 3‐phenylbenzofulvene nucleus. These interactions established among the chromophores apparently affected the photoisomerization mechanism and efficiency. The nanostructured aggregates (i. e. spherical nanoparticles) in water can represent an intermediate aggregation form of the chromophore that looks towards the well ordinate crystalline solid state and represents an interesting opportunity to characterize in depth the differences in photophysical and photochemical features between isolated molecules and aggregate species. The actual mechanism at the basis of this outstanding effect is at the present under computational and experimental investigations.

## Experimental Section

**X‐ray crystallography**: Single crystal of *E*‐**1** was submitted to X‐ray data collection on an Oxford‐Diffraction Xcalibur Sapphire 3 diffractometer with a graphite monochromated Mo–Kα radiation (*λ*=0.71073 Å) at 293 K. The structure was solved by direct methods implemented in SHELXS‐97 program.^[74]^ The refinement was carried out by full‐matrix anisotropic least squares on F2 for all reflections for non‐H atoms by means of the SHELXL program.^[75]^

Deposition Number 2071148 (for *E*‐**1**) contain the supplementary crystallographic data for this paper. These data are provided free of charge by the joint Cambridge Crystallographic Data Centre and Fachinformationszentrum Karlsruhe Access Structures service.

**Transient absorption (TA) spectroscopy**: TA experiments were performed with an experimental set‐up already described elsewhere[^38^, ^76^, ^77^] In short, an amplified Ti : Sa laser system (Amplitude) delivering 40 fs, 800 nm pulses at 5 kHz is used to pump a commercial optical parametric amplifier (TOPAS, Light conversion) followed by a frequency mixing stage in order to produce tunable pump pulses centered at λ_pu_=290 nm or λ_pu_=450 nm, with ∼60 to 80 fs duration. A few μJ of the fundamental 800‐nm pulse is used to generate a white light supercontinuum in CaF_2_, used as a probe pulse in the 310–700 nm range. The pump‐induced change of the sample absorbance ΔA is monitored by detecting the transmission of the probe pulse through the sample with a spectrograph offering a ∼300‐nm broad detection window. The data displayed in Figure 5 are the result of one experiment with λ_pu_=450 nm with the probing detection range tuned to λ_pr_=360–690 nm, and two experiments with λ_pu_=290 nm with probing detection windows of λ_pr_=360–690 nm and λ_pr_=300–400 nm (through UG11 color filter). Both datasets obtained with λ_pu_=290 nm are appended in one single dataset illustrated in Figure 5A. For each TA experiment performed on *E*‐**1** in solution, the same experiment is performed immediately after in the same conditions on the pure solvent in order to record and subtract solvent signal. In addition, the data displayed in Figure 5 panels A and B are also further processed to correct for the probe light dispersion (so‐called chirp) which results in a wavelength‐dependent time origin in the raw data (not shown).^[78]^ Both datasets are analyzed independently via global analysis^[79]^ consisting of singular value decomposition (data reduction) and global fit of the 3 dominating singular kinetic traces using a multiexponential decaying function convolved with a gaussian instrument response function (IRF), see for example ref [38, 77] for details about data analysis procedures. The result of the fit is displayed by plotting the decay‐associated spectra (DAS) which reveal the spectral dependence of the amplitude associated to each decay time of the multiexponential function. Positive (resp. negative) DAS means decaying (resp. rising) TA signal at the corresponding wavelengths.

**Quantum yield measurements**: The *trans*→*cis* photoisomerization quantum yield (φ_*trans→cis*_) of compound **1** was evaluated by collecting the absorption spectra while irradiating the *trans* isomer in methanol (concentration 5×10^−5^ M) at 320 nm. Negligible modifications were observed in the spectra (suggesting that the change in absorbance is lower than 0.02) during the 2 h (7200 s) of irradiation. In particular, the transformation was followed at 282 nm, where the difference in the molar absorption coefficients of the *trans* and *cis* isomers is the largest (Δϵ≈11000 M^−1^ cm^−1^). The xenon lamp emitted intensity at 320 nm was measured by potassium ferrioxalate actinometry and found to be 2.2×10^14^ photons s^−1^, corresponding to 3.65×10^−10^ Einstein s^−1^. An estimate of the photoisomerization quantum yield (φ_*trans→cis*_<7×10^−4^) was obtained according to the following equation:

$$\varphi =\frac{mol\;photoproduct}{mol\;photons}<\frac{\frac{0.02}{11000\;M^{-1}{cm}^{-1}\times 1\;cm}\times 10^{-3}L}{3.65\times 10^{-10}\;Einstein\;s^{-1}\times 7200\;s}={6.9\times 10}^{-4}$$

**Determination of CAC values by fluorescence analysis**: A stock solution of pyrene (6.0×10^−5^ M in acetone) was prepared and then aliquots of 5 μL were placed into vials and evaporated to remove acetone in an orbital shaker at 37 °C. Subsequently, 0.5 mL of compound **3** solution in bidistilled water, at concentrations ranging from 1×10^−5^ to 10 mg/mL were added to the pyrene residue; the final concentration of pyrene was 6.0×10^−7^ M in each sample. The solutions were kept at 37 °C for 24 h under continuous stirring to equilibrate pyrene with micelles. Pyrene emission spectra were recorded at 37 °C using an excitation wavelength of 333 nm. The CAC of the compound was calculated by plotting the I_373_/I_384_ (I_1_/I_3_) ratio, obtained from the emission spectra recorded at 37 °C, versus the logarithm of the compound concentration.

**DLS measurements**: DLS measurements were performed at 25 °C using a Malvern Zetasizer NanoZS instrument, fitted with a 532 nm laser at a fixed scattering angle of 173° with dispersions of **3** in bidistilled water.

**Transmission electron microscopy (TEM) experiments**: A drop of 3.5 μL of **3** solution in water was dropped onto a 300 mesh formvar‐coated copper grid. After 2 min, the excess of sample was blotted by filter paper and the grids were stained with 1 % of aqueous uranyl acetate solution. Samples were observed in a FEI Tecnai G2 Spirit transmission electron microscopy at an acceleration voltage of 100 kV.

**Cryo‐transmission electron microscopy (CryoTEM) experiments**: Vitrified specimens were prepared by adding 2.5 μL of compound 3 solution (10 mg/mL) to freshly plasma cleaned QUANTIFOIL®R 2/1 300‐mesh copper grids. A Vitrobot Mark IV (FEI) with 100 % humidity at 20 °C, a blot time of 3 s, the blot force set to −3, and a wait time of 2 s, was used to plunge freeze the grids in liquid ethane. The images were collected on CM 200 FEG (Philips) operating at 200 kV, equipped with a TVIPS F224HD CCD camera using TVIPS EM‐Menu software for image acquisition.

## Conflict of interest

The authors declare no conflict of interest.

1

## Supporting information

As a service to our authors and readers, this journal provides supporting information supplied by the authors. Such materials are peer reviewed and may be re‐organized for online delivery, but are not copy‐edited or typeset. Technical support issues arising from supporting information (other than missing files) should be addressed to the authors.

Supporting Information

## Data Availability

The data that support the findings of this study are available from the corresponding author upon reasonable request.
