# Supplementary material for: Design, Synthesis and Characterization of a Visible‐Light‐Sensitive Molecular Switch and Its PEGylation Towards a Self‐Assembling Molecule
Source: Chemistry. 2022 Jul 13;28(50):e202201477. doi: 10.1002/chem.202201477 (PMC9541190; doi:10.1002/chem.202201477)
Supplement: Supplementary file 1 — Supporting Information [file CHEM-28-0-s001.pdf]

Chemistry–A European Journal

Supporting Information

Design, Synthesis and Characterization of a Visible-Light-Sensitive Molecular Switch and Its PEGylation Towards a Self-Assembling Molecule

Marco Paolino,* Mario Saletti, Annalisa Reale, Mariano Licciardi, Paola Varvarà, Arnaud Marquette, Jérémie Léonard, Claudia Bonechi, Alessandro Donati, Gianluca Giorgi, Germano Giuliani, Benedetta Carlotti, Fausto Ortica, Loredana Latterini, Mariangela Gentile, Eugenio Paccagnini, Massimo Olivucci, and Andrea Cappelli*

Supporting Information

Synthesis

The synthetic work started from the preparation of molecular switch model **1** (Scheme S1) in order to evaluate the efficacy of the previously developed methodology (i. e. aldol reaction with *N*-Boc-pyrrolidinone and dehydration of the aldol intermediate) when applied to 3-phenylindenone-2-carboxylate **4**.[1]

Scheme S1. Synthesis of molecular photoswitch model **1**.

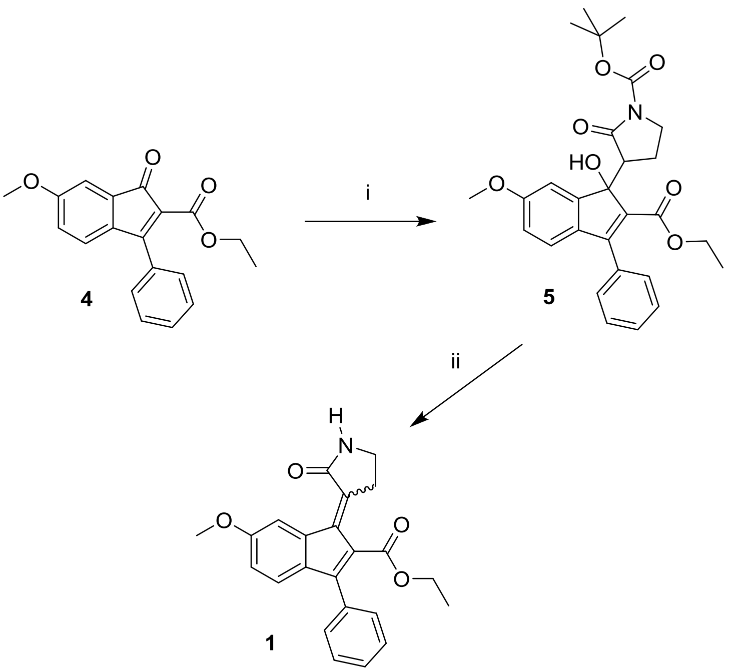

Reagents: (i) N-Boc-pyrrolidinone, LiHDMS, $BF_3(Et)_2O$, THF; (ii) PTSA, toluene.

As expected, the synthetic procedure was demonstrated to be robust enough and worked also with 3-phenylindenone-2-carboxylate **4** as the substrate. However, the dehydration of diastereomeric mixture of carbinol intermediate **5** required a careful optimization work and the use of *p*-toluensulfonic acid (PTSA) in refluxing toluene. In such a way, molecular switch

model **1** was obtained in acceptable yield (32%). Crystallographic studies performed on the red prisms obtained by recrystallization from ethyl acetate allowed us to assign the *E* geometry to the major isomer of compound **1** (**Figure 2**). On the other hand, the corresponding *Z* isomer was formed only in little amounts in the dehydration mixture confirming the existence of a thermostationary equilibrium strongly shifter through the *E* isomer in the reaction condition. However, as shown below, this equilibrium could be altered with the light allowing the isolation by chromatography of the *Z* isomer, which remains stable without interconverting at room temperature. A possible reason for the relatively low yields of the dehydration reaction appeared to be the formation of polymeric substances, which, owing to the presence of the benzofulvene structure in compound **1**, will be taken into consideration in next polymerization studies.

Based on the above results, the same synthetic methodology was used in the preparation of molecular switch **2** (Scheme S2), which was identified as potential functionalizable photoswitch.

Scheme S2. Synthesis of molecular photoswitch **2**.

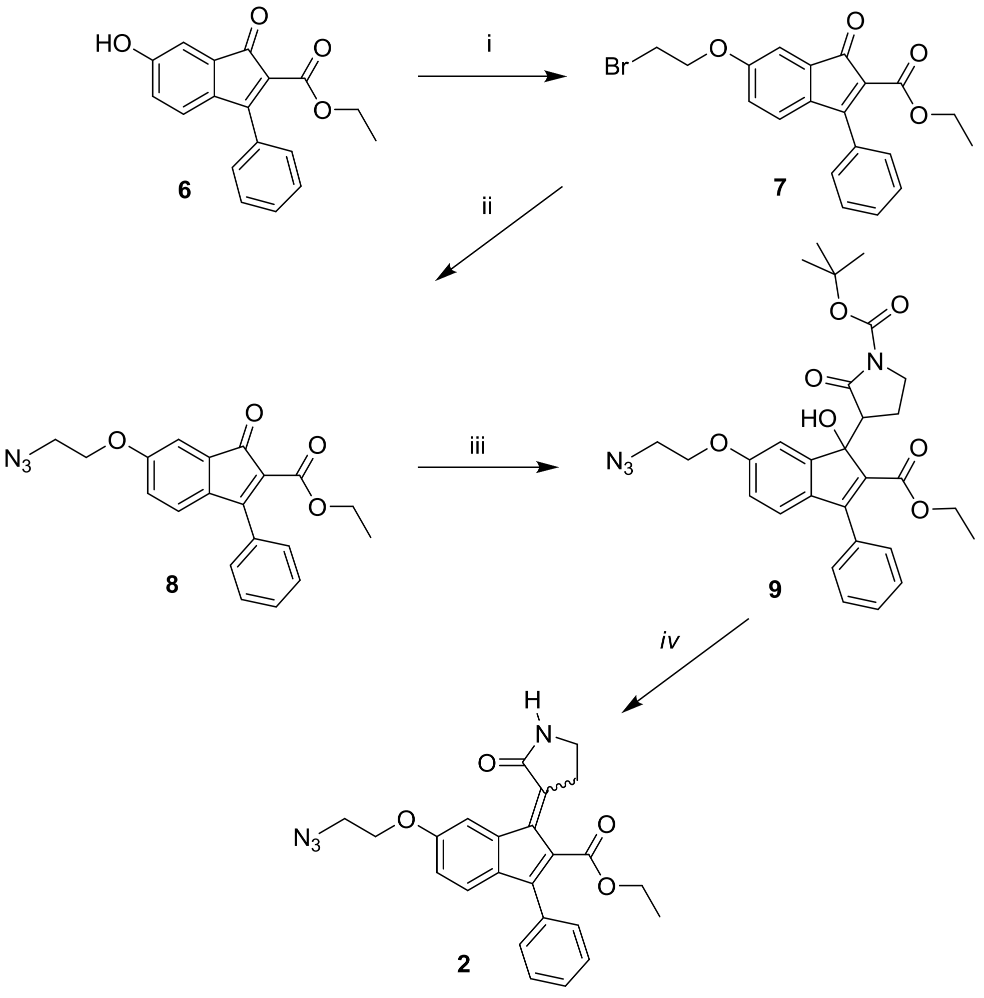

Reagents: 1,2-dibromoethane, K_2CO_3, DMF/CH_3CN; (ii) sodium azide, tetrabutylamonium bromide, DMF; (iii) N-Boc-pyrrolidinone, LiHDMS, $BF_3(Et)_2O$, THF; (iv) PTSA, toluene.

In particular, the phenol group of indenone derivative **6**[2] was alkylated with 1,2-dibromoethane in the presence of potassium carbonate as the base to obtain bromide **7**, which was reacted with sodium azide to give azide **8**. This latter was submitted to the aldol reaction-dehydration procedure to obtain the intermediate switch **2** ready to be functionalized with alkyne groups in a copper(1)-catalyzed azide alkyne 1,3-dipolar cycloaddition (CuAAC) reaction. In order to confirm the stability of this chromophore in the CuAAC reaction conditions and increase its affinity for polar solvents, compound **2** was conjugated with a methyl-end-capped nona(ethylene glycol) derivative bearing a propargyl group[3] at the other

end (Scheme S3) in DMF using CuBr as catalyst to obtain the water-soluble molecular switch **3**.

Scheme S3. Introduction of a solubilizing nona(ethylene glycol) side chain in molecular switch **2**.

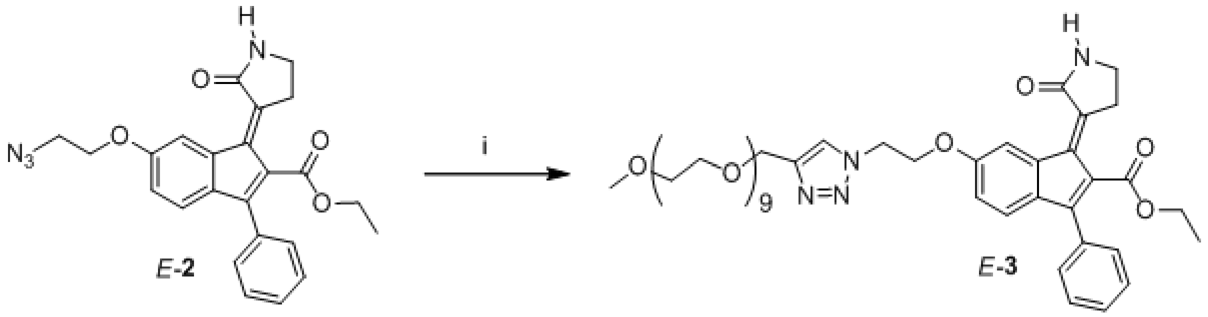

Reagents: (i) $CH_3O(C_2H_4O)_9CH_2CCH$, CuBr, DIPEA, DMF.

Thus, pure *E* isomer of **3** (i. e. *E*-**3**) was obtained from the corresponding isomer of starting **2**, and the structure was characterized by NMR analysis. Compound **3** was easily dissolved in the most common organic solvent such as dichloromethane, chloroform, methanol and also in water providing perfectly transparent red-orange solutions**.**

Materials and Methods.

Synthesis. All chemicals used were of reagent grade. Yields refer to purified products and are not optimized. Merck silica gel 60 (230−400 mesh) was used for column chromatography. Merck TLC plates and silica gel 60 F_{254} were used for TLC. NMR spectra were obtained with a Bruker DRX-400 AVANCE or a Bruker Avance III 500 spectrometer in the indicated solvents (TMS as internal standard). The values of the chemical shifts are expressed in ppm and the coupling constants (*J*) in Hz. An Agilent 1100 LC/MSD operating with an electrospray source was used in mass spectrometry experiments. The absorption spectra were recorded with a PerkinElmer Lambda 40 in the indicated solvent.

tert-Butyl 3-[2-(ethoxycarbonyl)-1-hydroxy-6-methoxy-3-phenyl-1*H*-inden-1-yl]-2-oxopyrrolidine-1-carboxylate (**5**).

To a solution of commercially available N-Boc-2-pyrrolidinone (0.44 g, 2.35 mmol) in anhydrous THF (15 mL), a 1 M solution of lithium hexamethyldisilazide (LiHMDS) in anhydrous THF (2.35 mL, 2.35 mmol) was added at −78 °C under a nitrogen atmosphere. After stirring for 15 min, a solution of compound **4** (0.60 g, 1.95 mmol) and $BF_3{\bullet}Et_2O$ (2.35 mmol, 290 μL) in anhydrous THF (15 mL) was added dropwise. The reaction mixture was stirred from −78 °C to room temperature in 4 h and then concentrated under reduced pressure. The residue was partitioned between a saturated solution of NH_4Cl and CH_2Cl_2. The organic layer was dried over Na_2SO_4 and concentrated under reduced pressure. Purification of the residue by flash chromatography with petroleum ether-ethyl acetate (7:3) as the eluent afforded the diastereomeric mixture of **5** (0.58 g, yield 60%) as a pale-yellow oil. ^{1}H NMR (400 MHz, $CDCl_3$): see Figure S4. MS(ESI): *m/z* 515.8 (M + Na^+).

Ethyl 6-methoxy-1-(2-oxopyrrolidin-3-ylidene)-3-phenyl-1*H*-indene-2-carboxylate (**1**).

A mixture of **5** (0.92 g, 1.86 mmol) in toluene (30 mL) containing *p*-toluensulfonic acid (PTSA) monohydrate (0.64 g, 3.37 mmol) was heated to reflux for 30 min. The reaction mixture was then concentrated under reduced pressure and the residue was partitioned between a saturated solution of $NaHCO_3$ and CH_2Cl_2. The organic layer was dried over sodium sulfate and concentrated under reduced pressure and the resulting residue was purified by flash chromatography on silica gel (9:1, ethyl acetate-petroleum ether as the eluent) to obtain compound **1** (0.22 g, yield 32%) showing *E* geometry at the exocyclic double bond, as suggested by the crystallographic studies performed on the red prisms (mp 187.1-190.0 °C) obtained by recrystallization from ethyl acetate by slow evaporation of the solvent. ^{1}H NMR (400 MHz, $CDCl_3$): 1.05 (t, J = 7.2, 3H), 3.29 (t, J = 6.2, 2H), 3.52 (t, J = 6.2, 2H), 3.86 (s,

3H), 4.09 (q, *J* = 7.1, 2H), 6.01 (br s, 1H), 6.75 (dd, *J* = 8.3, 2.4, 1H), 7.04 (d, *J* = 8.3, 1H), 7.32-7.45 (m, 5H), 9.06 (d, *J* = 2.2, 1H). ^{13}C NMR (150 MHz, $CDCl_3$): 13.8, 29.9, 40.1, 55.6, 61.2, 114.3, 115.2, 122.2, 128.2, 128.3, 128.6, 128.7, 133.4, 134.3, 136.3, 141.3, 151.4, 161.0, 168.0, 171.2. MS(ESI): *m/z* 398.1 (M + Na^+) (Figure S5).

The pure *Z* isomer was obtained by irradiation at 440 nm until to reach the PSS and further purification by flash chromatography on silica gel (9:1, ethyl acetate-petroleum ether as the eluent) as red solid (mp 176.4-179.3 °C). ^{1}H NMR (400 MHz, $CDCl_3$): 1.09 (t, *J* = 7.2, 3H), 3.46 (t, *J* = 6.0, 2H), 3.64 (t, *J* = 6.0, 2H), 3.84 (s, 3H), 4.13 (q, *J* = 7.1, 2H), 6.26 (br s, 1H), 6.76 (dd, *J* = 8.3, 2.2, 1H), 7.09 (d, *J* = 2.0, 1H), 7.13 (d, *J* = 8.3, 1H), 7.39 (m, 3H), 7.50 (m, 2H).

Ethyl 6-(2-bromoethoxy)-1-oxo-3-phenyl-1*H*-indene-2-carboxylate (**7**).

A mixture of hydroxyindenone derivative **6**[4] (3.24 g, 11 mmol) in DMF-CH_3CN (1:1, 40 mL) containing K_2CO_3 (6.2 g, 44.9 mmol) and 1,2-dibromoethane (4.82 mL, 55.7 mmol) was heated to reflux under vigorous stirring for 2 h. The solid was then filtered off and the filtrate was concentrated under reduced pressure. The resulting residue was partitioned between a saturated solution of NH_4Cl and CH_2Cl_2. The organic layer was dried over sodium sulfate and concentrated under reduced pressure and the residue was purified by flash chromatography with petroleum ether-ethyl acetate (8:2) as the eluent to give compound **7** as a red crystalline solid (2.75 g, yield 62%, mp 123.4-123.9 °C). ^{1}H NMR (400 MHz, $CDCl_3$): 1.14 (t, *J* = 7.1, 3H), 3.63 (t, *J* = 6.2, 2H), 4.17 (q, *J* = 7.1, 2H), 4.33 (t, *J* = 6.1, 2H), 6.85 (dd, *J* = 8.1, 2.2, 1H), 7.08 (d, *J* = 8.2, 1H), 7.17 (d, *J* = 2.1, 1H), 7.49 (s, 5H). MS(ESI): *m/z* 422.7, 424.6 (M + Na^+).

Ethyl 6-(2-azidoethoxy)-1-oxo-3-phenyl-1*H*-indene-2-carboxylate (**8**).

A mixture of bromide derivative **7** (0.56 g, 1.396 mmol) in DMF (10 mL) containing tetrabutylammonium bromide (0.018 g, 0.056 mmol) and sodium azide (0.36 g, 5.54 mmol) was stirred at room temperature for 60 h and then concentrated under reduced pressure. The resulting oily residue was dissolved into CH_2Cl_2, dried over sodium sulfate, and the solvent was removed under reduced pressure. Purification of the residue by flash chromatography with petroleum ether-ethyl acetate (8:2) as the eluent afforded compound **8** as a red crystalline solid (0.47 g, yield 93%, mp 108.5-108.8 °C). ^{1}H NMR (400 MHz, DMSO-d_6): 1.03 (t, J = 7.1, 3H), 3.65 (t, J = 4.7, 2H), 4.06 (q, J = 7.0, 2H), 4.27 (t, J = 4.7, 2H), 7.03 (dd, J = 8.2, 2.4, 1H), 7.14 (d, J = 2.2, 1H), 7.17 (d, J = 8.2, 1H), 7.54 (m, 5H). MS(ESI): *m/z* 385.9 (M + Na^+).

tert-Butyl 3-[6-(2-azidoethoxy)-2-(ethoxycarbonyl)-1-hydroxy-3-phenyl-1*H*-inden-1-yl]-2-oxopyrrolidine-1-carboxylate (**9**).

To a solution of N-Boc-2-pyrrolidinone (0.17 g, 0.918 mmol) in anhydrous THF (7.0 mL), a 1 M solution of lithium hexamethyldisilazide (LiHMDS) in anhydrous THF (0.91 mL, 0.91 mmol) was added at −78 °C under a nitrogen atmosphere. After stirring for 15 min, a solution of compound **8** (0.28 g, 0.77 mmol) and $BF_3{\bullet}Et_2O$ (110 μL, 0.91 mmol) in anhydrous THF (7.0 mL) was added dropwise. The resulting mixture was stirred from −78 °C to room temperature in 4 h and then concentrated under reduced pressure and the residue was partitioned between a saturated solution of NH_4Cl and CH_2Cl_2. The organic layer was dried over Na_2SO_4 and concentrated under reduced pressure. Purification of the residue by flash chromatography with petroleum ether-ethyl acetate (7:3) as the eluent gave a diastereomeric mixture of **9** (0.24 g, yield 57%) as a pale yellow solid. ^{1}H NMR (400 MHz, $CDCl_3$): see Figure S6. MS(ESI): *m/z* 570.8 (M + Na^+).

Ethyl (*E*)-6-(2-azidoethoxy)-1-(2-oxopyrrolidin-3-ylidene)-3-phenyl-1*H*-indene-2-carboxylate (**2**).

A mixture of indenol derivative **9** (0.35 g, 0.638 mmol) in toluene (150 mL) containing PTSA monohydrate (0.55 g, 2.89 mmol) was heated to reflux for 30 min, and then concentrated under reduced pressure. The residue was partitioned between a saturated solution of $NaHCO_3$ and CH_2Cl_2 and the organic layer was dried over sodium sulfate and concentrated under reduced pressure. Purification of the resulting residue by flash chromatography ethyl acetate-petroleum ether (9:1) as the eluent afforded compound **2** (0.070 g, yield 25%) showing *E* geometry at the exocyclic double bond. An analytical sample was prepared by recrystallization from ethyl acetate by slow evaporation to obtain yellow needles melting at 128.3-128.9 °C. ^{1}H NMR (400 MHz, $CDCl_3$): 1.05 (t, *J* = 7.2, 3H), 3.29 (t, *J* = 6.1, 2H), 3.53 (t, *J* = 6.1, 2H), 3.60 (t, *J* = 5.0, 2H), 4.10 (q, *J* = 7.1, 2H), 4.24 (t, *J* = 5.0, 2H), 6.28 (br s, 1H), 6.79 (dd, *J* = 8.3, 2.4, 1H), 7.05 (d, *J* = 8.3, 1H), 7.33-7.55 (m, 5H), 9.10 (d, *J* = 2.3, 1H). ^{13}C NMR (150 MHz, $CDCl_3$): 13.7, 29.3, 40.2, 50.2, 61.2, 67.2, 115.3, 115.5, 122.3, 128.2, 128.4, 128.8, 129.0, 133.3, 134.9, 136.2, 141.2, 151.3, 159.5, 167.9, 171.3. MS(ESI): *m/z* 453.1 (M + Na^+) (Figure S7).

Ethyl (*E*)-6-[2-[4-(2,5,8,11,14,17,20,23,26,29-decaoxatriacontyl)-1*H*-1,2,3-triazol-1-yl]ethoxy]-1-(2-oxopyrrolidin-3-ylidene)-3-phenyl-1H-indene-2-carboxylate (**3**).

A mixture of **2** (100 mg, 0.23 mmol) in DMF (5.0 mL) containing $CH_3O(C_2H_4O)_9CH_2CCH$[3] (170 mg, 0.36 mmol), copper(I) bromide (17 mg, 0.12 mmol), and DIPEA (40 microL, 0.24 mmol) was exposed to MW irradiation into a CEM Discover apparatus (5 min, T = 60 °C, 250 W) and then diluted with dichloromethane and washed with a saturated solution of NH_4Cl. The organic layer was dried over Na_2SO_4 and concentrated under reduced pressure. Purification of the residue by flash chromatography with dichloromethane-methanol (9:1) as the eluent afforded compound 3 as an red-orange oil (160 mg, yield 78%). ^{1}H NMR (400

MHz, $CDCl_3$): 1.05 (t, *J* = 6.9, 3H), 3.28 (t, *J* = 6.1, 2H), 3.36 (s, 3H), 3.52 (m, 2H), 3.56-3.75 (m, 36H), 4.10 (q, *J* = 7.1, 2H), 4.43 (t, *J* = 5.0, 2H), 4.68 (s, 2H), 4.76 (t, *J* = 4.9, 2H), 6.17 (br s, 1H), 6.73 (d, *J* = 8.2, 1H), 7.04 (d, *J* = 8.2, 1H), 7.42 (m, 5H), 7.84 (s, 1H), 9.11 (s, 1H). ^{1}H NMR (400 MHz, CD_3OD): 1.06 (t, *J* = 6.9, 3H), 3.22 (t, *J* = 5.8, 2H), 3.32 (s, 3H), 3.48-3.64 (m, 38H), 4.10 (q, *J* = 7.3, 2H), 4.45 (t, *J* = 4.8, 2H), 4.63 (s, 2H), 4.82 (m, overlapped with H_2O, 2H), 6.79 (d, *J* = 8.4, 1H), 7.03 (d, *J* = 8.3, 1H), 7.30-7.54 (m, 5H), 8.11 (br s, 1H), 8.98 (s, 1H). ^{13}C NMR (150 MHz, $CDCl_3$): 13.7, 30.0, 39.8, 50.8, 59.0, 61.2, 63.7, 66.5, 70.1, 70.5, 72.0, 115.2, 115.9, 122.1, 125.2, 128.2, 128.4, 128.7, 129.4, 133.2, 135.4, 136.3, 140.2, 144.8, 150.5, 158.8, 168.0, 170.0. ^{13}C NMR (150 MHz, CD_3OD): 14.0, 31.0, 40.9, 51.0, 59.0, 62.5, 64.9, 67.8, 70.6, 71.3, 71.5, 72.9, 115.4, 117.3, 123.0, 126.0, 129.3, 129.5, 129.9, 130.8, 134.5, 136.3, 137.4, 138.4, 140.8, 146.2, 150.9, 160.5, 169.6, 172.5. MS(ESI): *m/z* 919.4 (M + Na^+) (Figure S8).

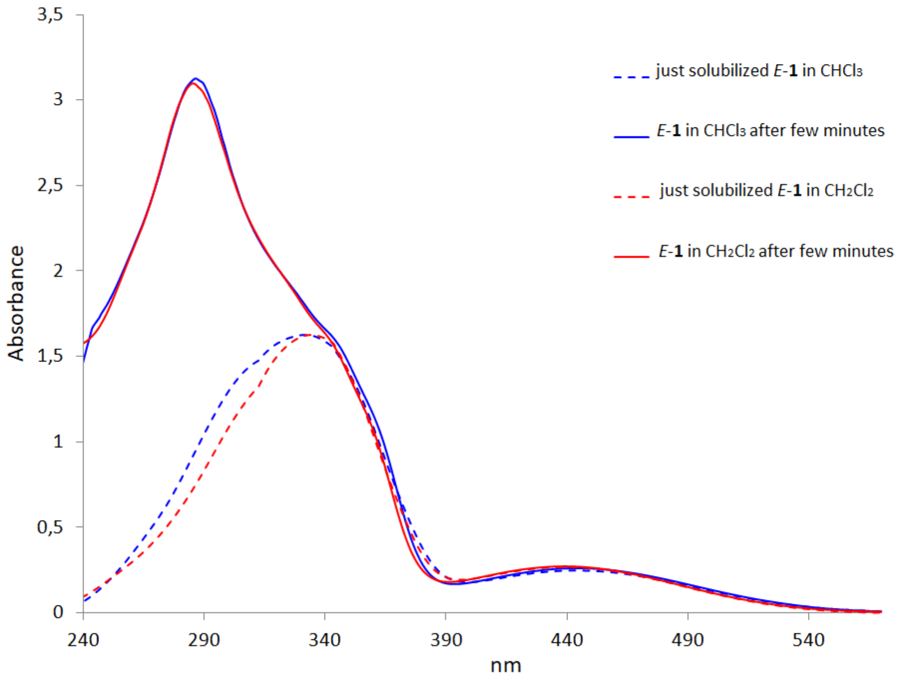

Figure S1. UV-vis absorption spectra of the prototype *E*-**1** in both chloroform (blue lines) and dichloromethane (red lines) at concentration of 0.02 mM recorded immediately after the apparent solubilization (dashed lines) and after a few minutes (continuos lines).

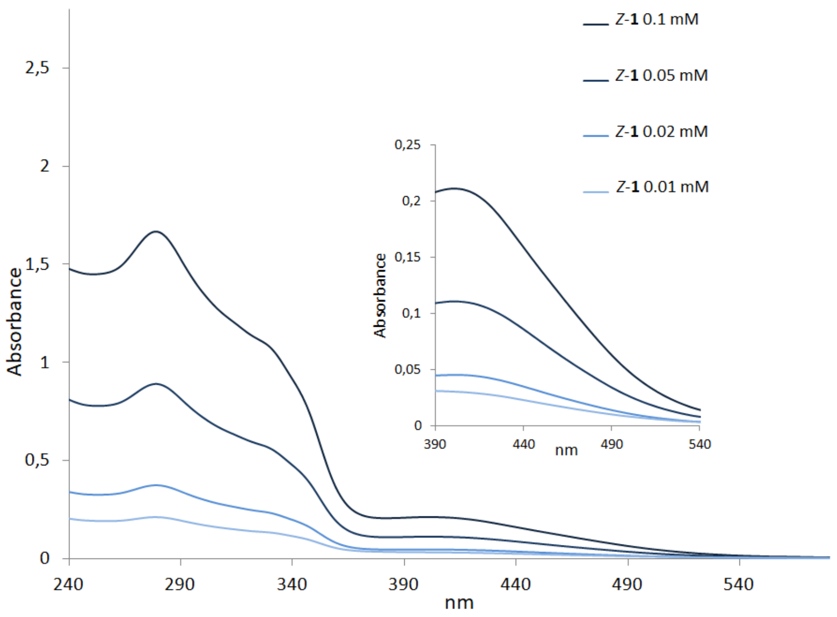

Figure S2. UV-vis absorption spectra of the prototype *Z*-**1** in methanol at different concentrations (from 0.1 to 0.01 mM). The visible region of the spectra has been highlighted in the box.

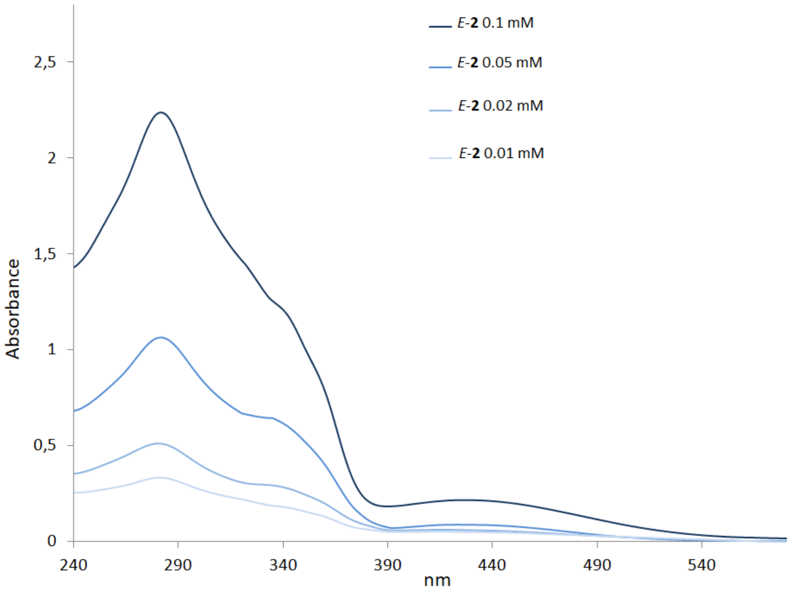

Figure S3. UV-vis absorption and spectra of *E*-**2** in methanol at different concentrations (from 0.1 to 0.01 mM).

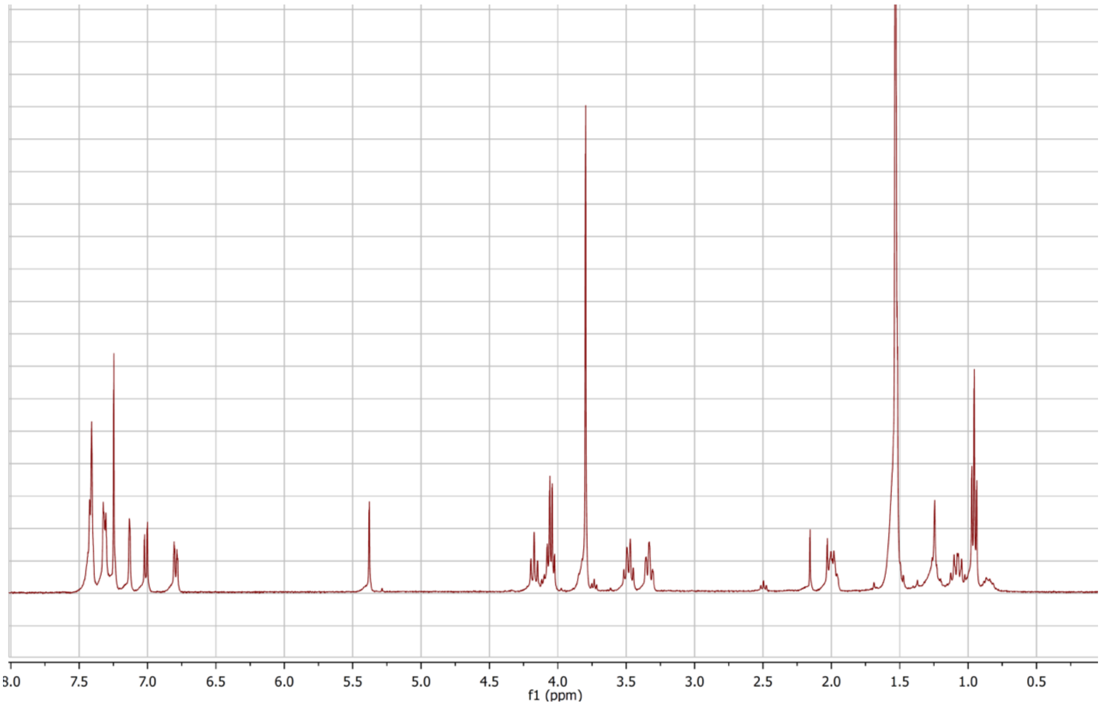

Figure S4. ^{1}H NMR spectrum (400 MHz, $CDCl_3$) of the diasteromeric mixture of **5**.

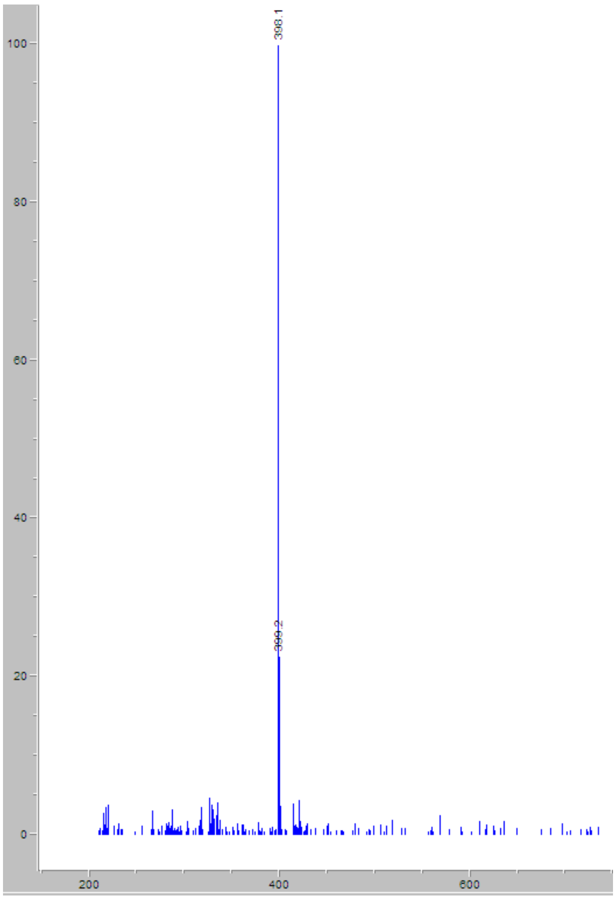

Figure S5. Mass spectrum (ESI) of compound **1**.

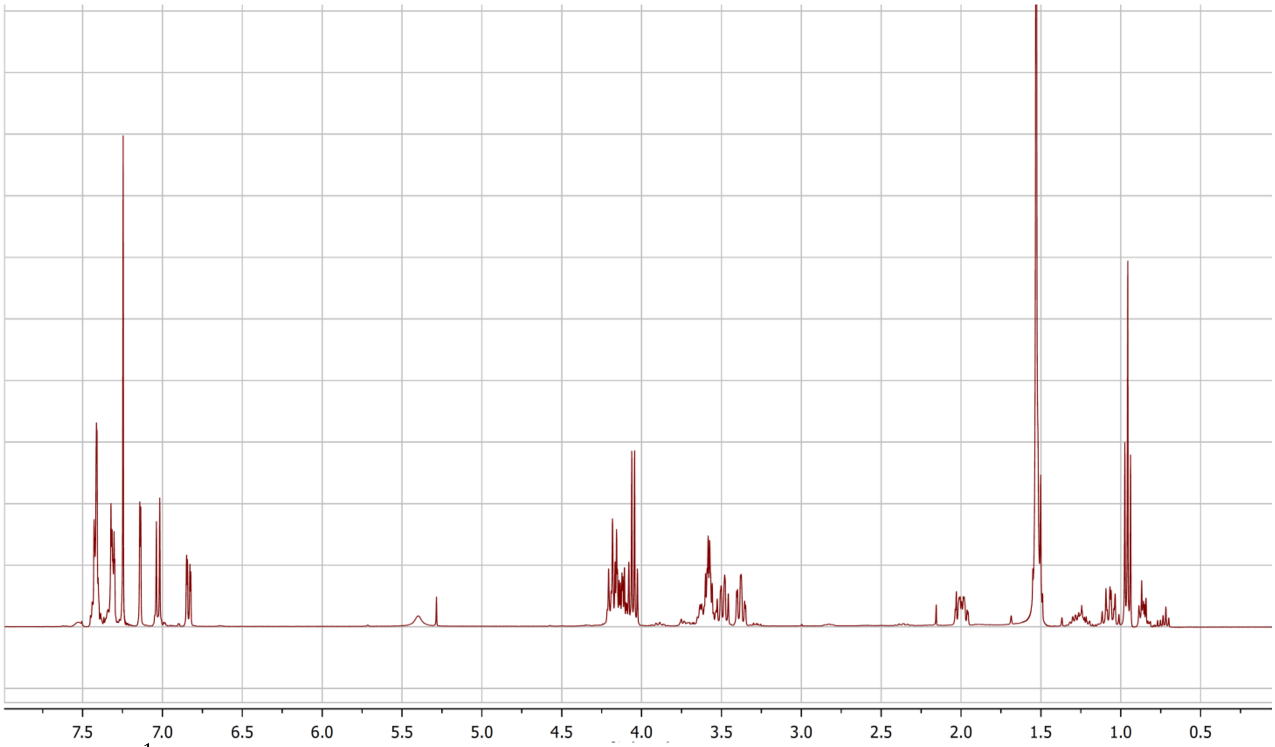

Figure S6. ^{1}H NMR spectrum (400 MHz, $CDCl_3$) of the diasteromeric mixture of **8**.

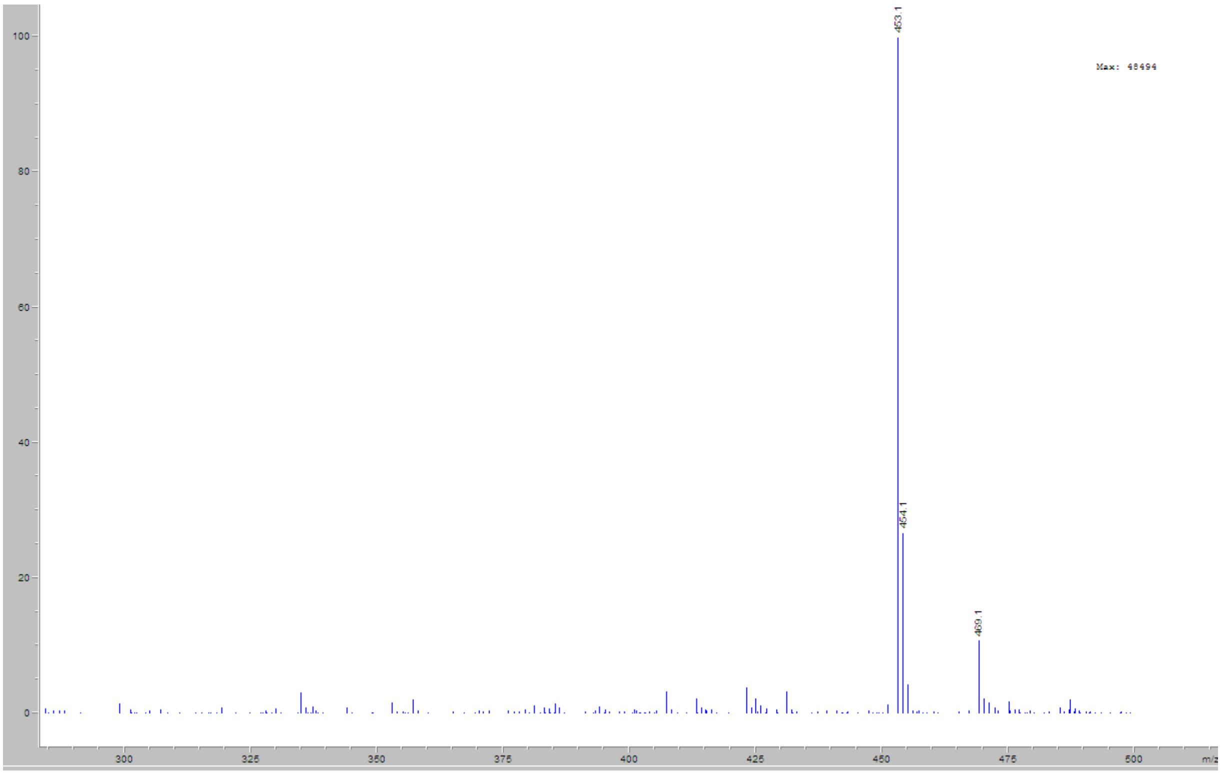

Figure S7. Mass spectrum of compound **2**.

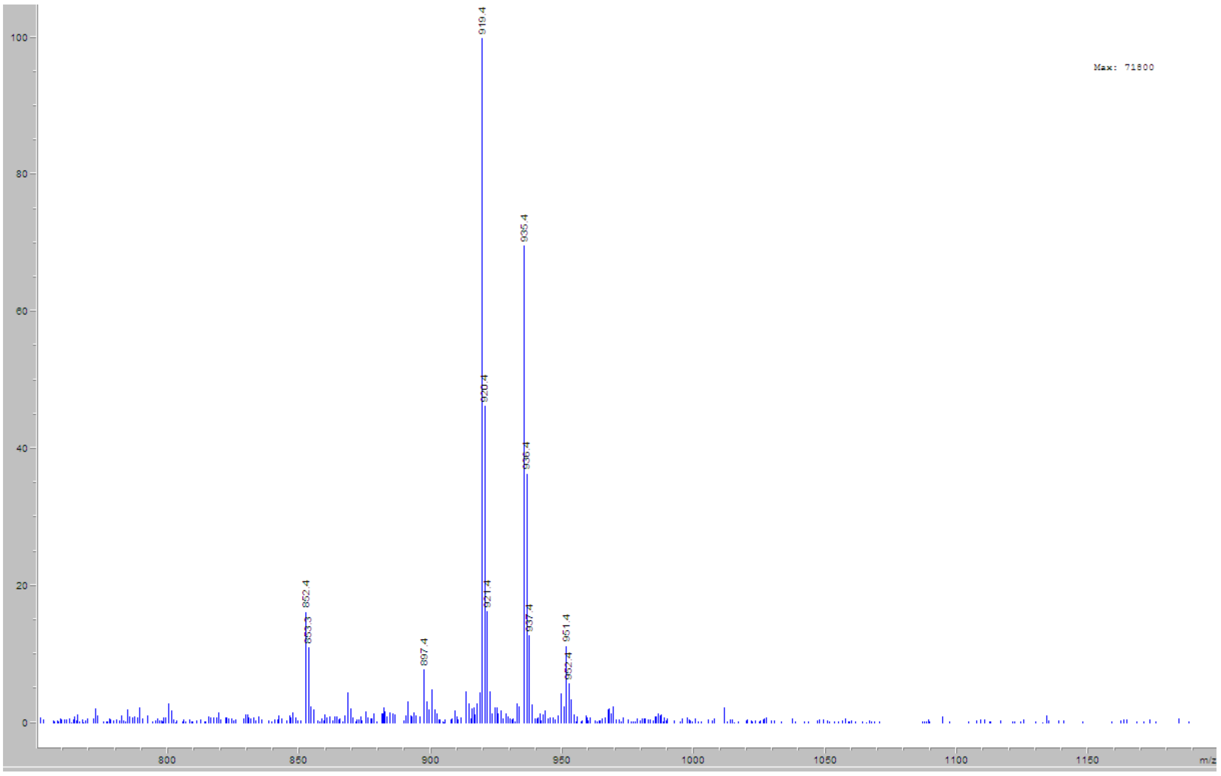

Figure S8. Mass spectrum of compound **3**.

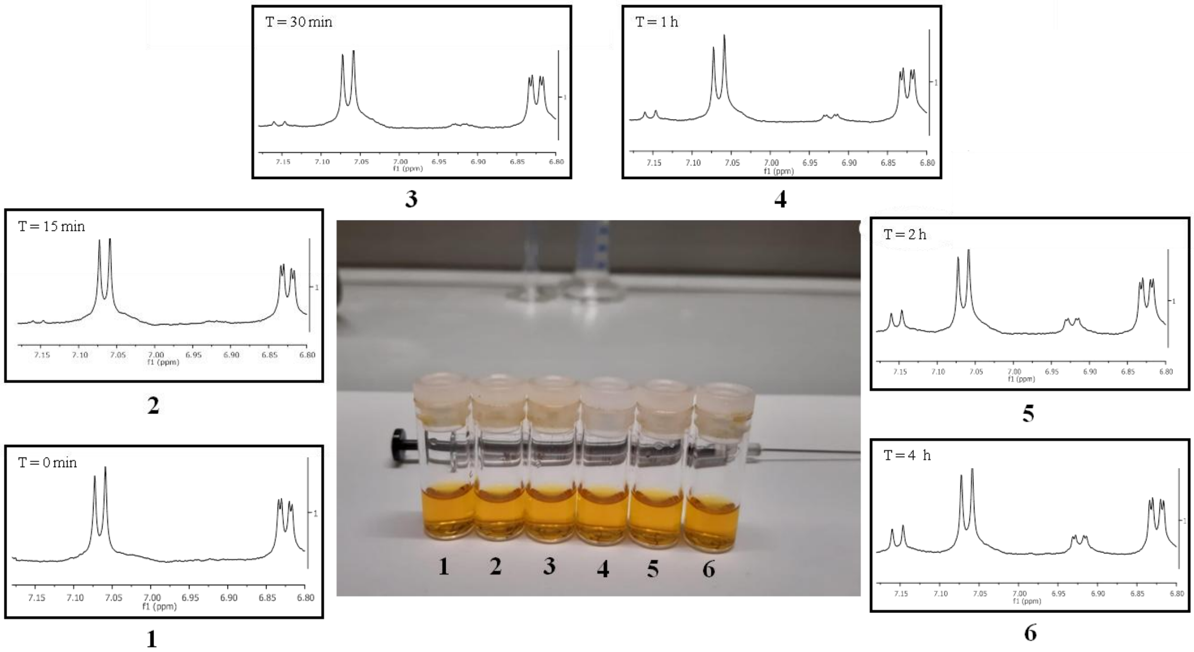

Figure S9. Progressive increase of the *Z*-**1** from a solution of *E*-**1** in deuterated methanol (0.001 M) as result of increasing irradiation times with UV-B light (2 x GT15T8 Hg tube 15 Watt).

References

[1] M. Paolino, M. Gueye, E. Pieri, M. Manathunga, S. Fusi, A. Cappelli, L. Latterini, D. Pannacci, M. Filatov, J. Léonard, et al., *J. Am. Chem. Soc.* **2016**, *138*, 9807–9825.

[2] A. Cappelli, M. Paolino, P. Anzini, G. Giuliani, S. Valenti, M. Aggravi, A. Donati, R. Mendichi, L. Zetta, A. C. Boccia, et al., *J. Polym. Sci. Part A Polym. Chem.* **2010**, *48*, 2446–2461.

[3] R. Shen, X. Shen, Z. Zhang, Y. Li, S. Liu, H. Liu, *J. Am. Chem. Soc.* **2010**, *132*, 8627–8634.

[4] A. Cappelli, M. Paolino, P. Anzini, G. Giuliani, S. Valenti, M. Aggravi, A. Donati, R. Mendichi, L. Zetta, A. C. Boccia, et al., *J. Polym. Sci. Part A Polym. Chem.* **2010**, *48*, 2446–2461.
